# Targeting T follicular helper cells to support lifelong health

**DOI:** 10.1038/s41573-026-01495-3

**Published:** 2026-07-24

**Authors:** Michelle A. Linterman, Di Yu, Louise M.C. Webb, Carola G. Vinuesa

**Affiliations:** 1 Malaghan Institute for Medical Research, Aotearoa New Zealand; 2 Immunology Programme, Babraham Institute, United Kingdom; 3 Frazer Institute, Faculty of Health, Medicine and Behaviour Sciences, The University of Queensland, Brisbane, Queensland, Australia; 4 Ian Frazer for Children’s Immunotherapy Research, Children’s Health Research Centre, Faculty of Health, Medicine and Behaviour Sciences, The University of Queensland, Brisbane , Queensland The University of Queensland, Brisbane , Queensland, Australia; 5 Francis Crick Institute, United Kingdom

## Abstract

T follicular helper (Tfh) cells support B cell function by promoting memory B cell differentiation and sustaining long-lasting antibody responses, thereby enabling immunity that protects the host from subsequent infections. The effectiveness of antibody mediated immunity via vaccination, has had a profound global impact in reducing the morbidity and mortality associated with infectious diseases, second only to the public health benefits of clean water and adequate sanitation. However, when antibodies are directed against self or non-pathogenic antigens, they can contribute to the development of autoimmune diseases, allergic reactions and transplant rejection. Tfh cells are essential for orchestrating antibody responses, providing help to B cells both within and outside germinal centres, and delivering key signals that drive immunoglobulin class switching and affinity maturation. Tfh cell formation and function is dynamic and highly adaptable with significant cellular plasticity to ensure robust antibody production accompanies most immune responses. Beyond supporting antibody responses, Tfh cells have also been implicated in cancer, diabetes, and atherosclerosis. Given their multifaceted roles in health and disease, and Tfh biology changing during normal ageing, the development of targeted strategies to modulate their activity remains an attractive approach to promote health across the lifespan.

## Introduction

**Figure 1:**
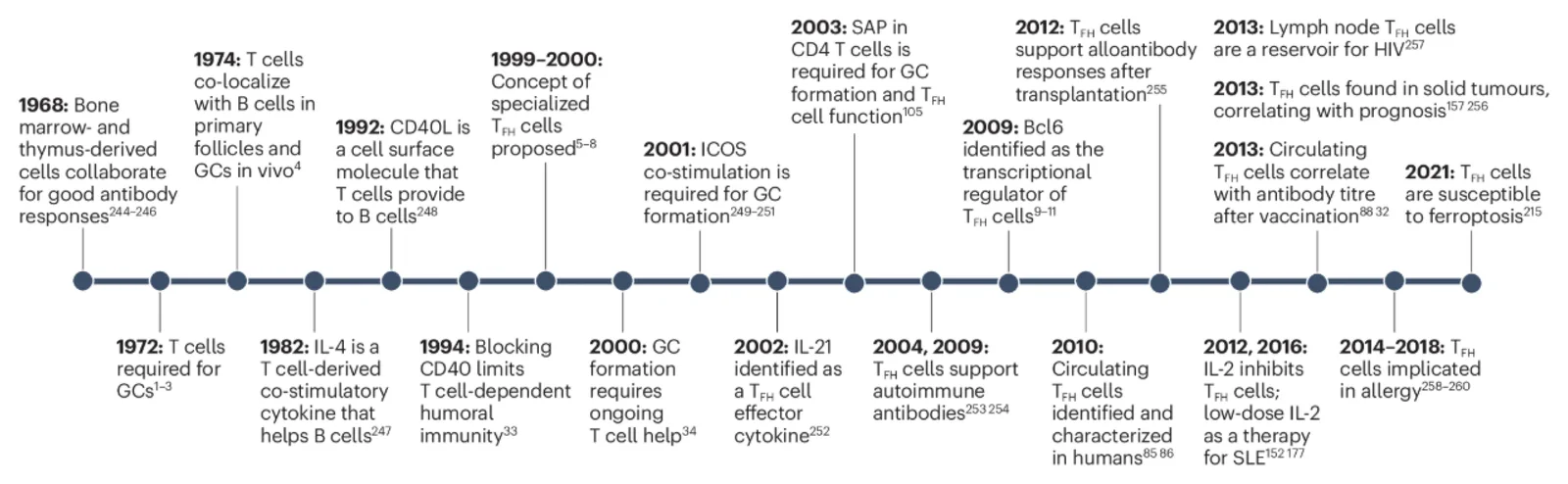
Early findings of T and B cells collaborating in germinal centres (GCs) led to the concept of T follicular helper (TFH) cells being proposed in 1999, and to the demonstration of their importance244,245,246,247,248,249,250,251,252,253,254,255,256,257,258,259,260 in vaccination, transplantation and various diseases. cT_FH_ cells, circulating T_FH_ cells; SLE, systemic lupus erythematosus.

It has long been recognised that humoral immunity benefits from T cell help (Figure 1). Early experiments showed that the reduced class-switched serum antibody titres and lack of germinal centres in athymic mice and humans could be rescued by transfer of thymocytes ^1, 2, 3^. Within secondary lymphoid organs, T and B cells are largely compartmentalised into distinct areas, with only rare T cells observed in primary B cell follicles, and T cells seen at higher numbers in the secondary follicles that contain germinal centres ^4^. This suggested that the support T cells provide to B cells must be provided at the interface of the T and B cell regions and/or driven by the few T cells found within B cell follicles. Two key findings created step changes in our understanding of T cell dependent humoral immunity and ignited research on T follicular helper (Tfh) cells: (1) The discovery that CXCR5-expressing helper T cells localised towards B cell areas and provided superior B cell help ^5, 6, 7, 8^, and (2) the identification of Bcl6 as the transcription factor required for their formation ^9, 10, 11^.

Like other CD4^+^ T cell subsets, an important feature of Tfh cells is their adaptability, shaping their response to fine tune the type of help they provide B cells as well as altering their location enabling targeted help ^12, 13, 14, 15, 16^. Viewed in the context of evolution, it is plausible that organisms have developed humoral immunity to complement the type of cellular immunity generated, enabling a flexible and adaptable response to infection that maximises protection against reinfection. This heterogeneity is not simply limited to cytokine production in response to different pathogen types; it also manifests across the organismal lifespan shaped by ageing and the response to different environmental factors such as the microbiome, hormones, dietary nutrients, and many other factors. Responsiveness of Tfh cell homeostasis to external cues provides an opportunity to target these cells to support health throughout the lifespan^17^.

We now understand the name “Tfh cells” to generally refer to T cells that have the functional capacity to provide help to B cells in secondary lymphoid tissues, whether that be in germinal centres or at the interface of the T and B cell zones ^12^. Typically, this response occurs in secondary lymphoid tissues but is also evident in tertiary lymphoid structures within inflamed non-lymphoid tissues. Both Tfh and Tfh-like cells have been described as important B cell helpers within these structures ^18, 19, 20^. T peripheral helper (Tph) cells have also been described at these sites; although they do not express CXCR5 or Bcl6 ^19, 21^, it is plausible they derive from Bcl6-expressing Tfh cells that have left secondary lymphoid tissues. Here, we will review fundamental Tfh cell function in vaccination, infection and in non-infectious disease, with Tph and T follicular regulatory cells being beyond the scope of this review. We will discuss known therapies that influence Tfh cells, and how recent advances in understanding fundamental Tfh cell biology provides new opportunities to develop therapeutics that target them.

### Tfh cell differentiation and function

Tfh cell differentiation initiates when naïve T cells are primed by antigen presenting cells that provide cognate antigenic and co-stimulatory signals. It is thought that type 2 conventional dendritic cells (cDC2) are the predominant antigen presenting cell initiating Tfh cell differentiation ^22, 23^. However, the type, dose and location of the antigenic stimulus that antigen presenting cells are responding to matters, since under different conditions, monocyte derived dendritic cells, Langerhans cells, type 1 conventional dendritic cells (cDC1) and even B cells can support the first steps of Tfh cell differentiation ^14, 24^. Within secondary lymphoid tissues, antigen presenting cell priming drives activated CD4^+^ T cells to relocate to the interface of the T and B cell areas by upregulating CXCR5 and EBI2. Here T cells can interact with activated dendritic cells and B cells ^25^. Cognate interactions with B cells complete Tfh cell differentiation, resulting in stabilised expression of Bcl6, the essential transcription factor for Tfh cell identity ^9, 10, 11, 26, 27, 28^.

**Figure 2:**
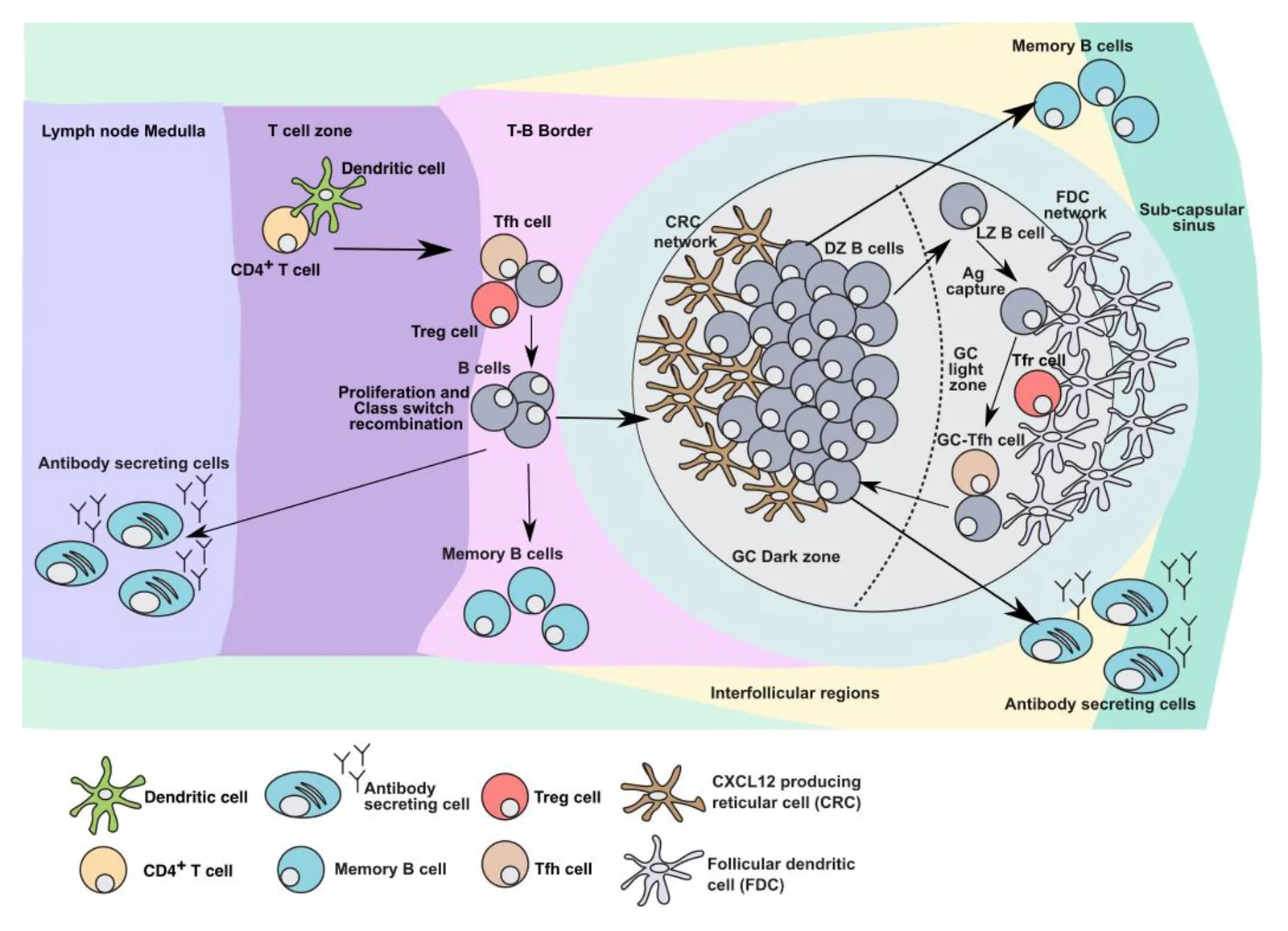
Tfh cells support B cell responses in secondary lymphoid tissues. After T cell priming in the T cell zone, a proportion of CD4+ helper T cells migrate to the T-B border. Here, these primed CD4+ helper T cells receive further differentiation cues from B cells to solidify their identity as Tfh cells. Tfh cells can provide help, in the form of cytokines and costimulatory receptors, to B cells to support proliferation, class-switch recombination and differentiation. After interaction with Tfh cells at the T:B border or interfollicular regions B cells can become memory B cells, antibody secreting cells or enter the follicle and become germinal centre (GC) precursors. GC dark zone B cells proliferate undergo somatic hypermutation of the genes encoding their B cell receptors. B cells are retained in the dark zone through CXCR4-dependent localisation to the CXCL12 producing reticular cell (CRC) network. By downregulating CXCR4 B cells then migrate from the dark zone to the light zone where they bind antigen held on follicular dendritic cells (FDCs) and then present this antigen on MHCII to Tfh cells. Culmination of successful interactions leads to production of memory B cells and antibody secreting cells.

Tfh : B cell cognate interactions facilitate the reciprocal exchange of cytokines, such as IL-21 and IL-4 secreted by Tfh cells, and co-stimulatory signals including CD40L from Tfh cells, and ICOSL from B cells which shape the emerging immune response. These signals are integrated at different stages and locations to facilitate class-switch recombination and the differentiation into antibody secreting cells, memory B cells and memory Tfh cells (Figure 2) ^29, 30, 31^. Tfh cell help is not an absolute requirement for B cell differentiation against non-protein antigens. Strong B cell receptor signals that crosslink multiple BCRs and innate costimulatory signals that activate Toll like receptors (TLRs) can be sufficient to support some antibody production, class switching, germinal centre formation, and differentiation; however, formation of persistent germinal centres is strictly dependent on Tfh cell help ^32, 33, 34^.

**Figure 3:**
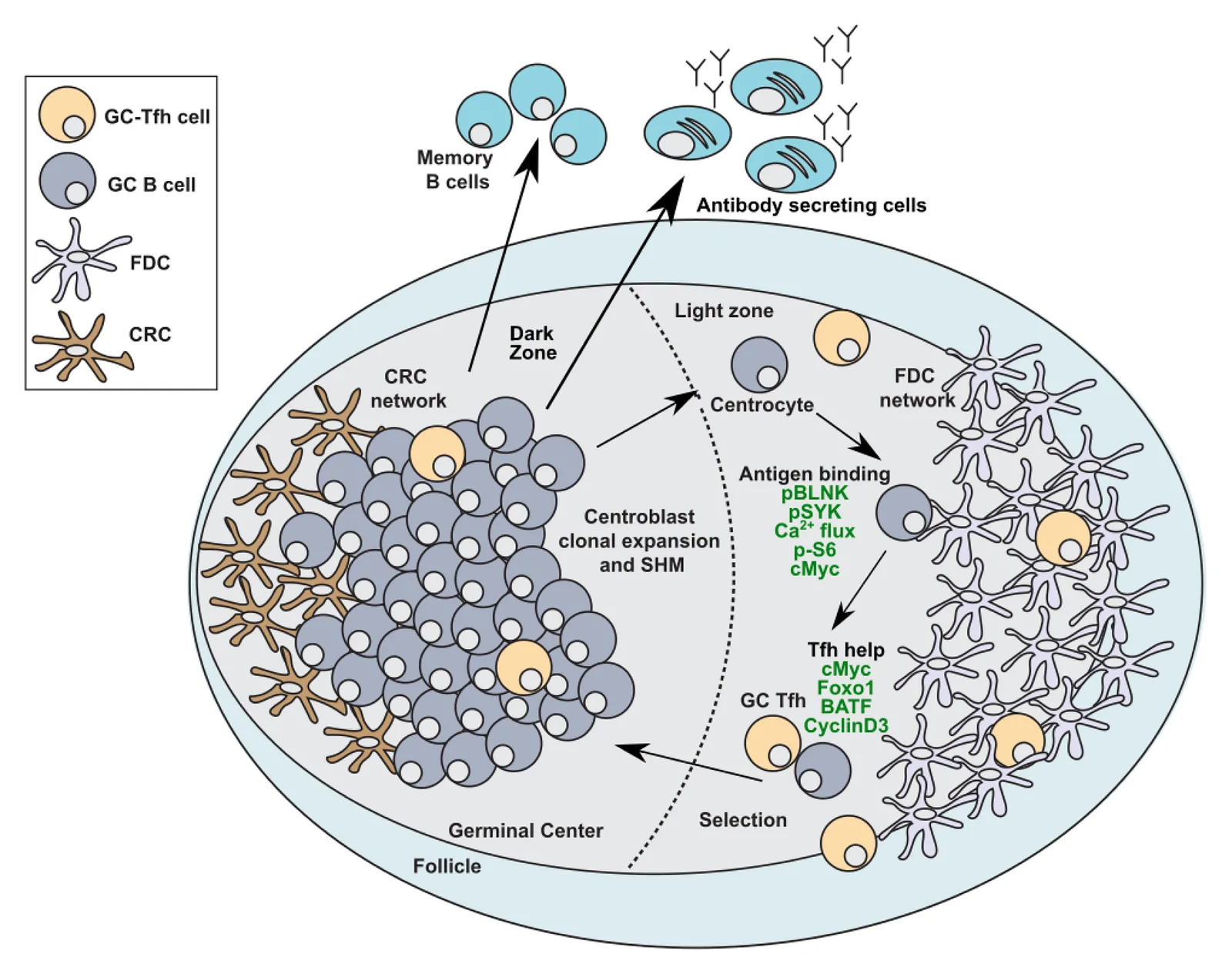
GC-Tfh cells support the continued proliferation of germinal centre B cells in the dark zone. After proliferation and somatic hypermutation (SHM) in the dark zone, B cells migrate to the light zone where their mutated and re-expressed B cell receptor binds antigen held on FDC. B cells with a BCR that is functional and specific receive BCR signaling resulting in Calcium signaling, phosphorylation of BLNK, SYK and S6 ribosomal protein, and cMyc upregulation. B cells that have a functional BCR then endocytose antigen obtained from FDCs and process this and present peptide:MHCII complexes to Tfh cells, enabling cognate interactions in which Tfh cells who provide CD40L, IL-21 and IL-4 to B cells. A B cell that receives T cell help increases expression of cMyc, Foxo1, BATF and Cyclin D3 to support further proliferation in the dark zone following CXCR4 dependent migration to this site in response to CXCL12 producing reticular cells (CRC).

GCs are inducible microenvironments that can be anatomically divided into functionally distinct zones. B cells undergo proliferation and somatic hypermutation in the dark zone before migrating to the light zone, where they sample antigen held on follicular dendritic cells, process it, and then present it to Tfh cells to elicit T cell help ^35^. Only a small proportion of Tfh cells are found in GCs. These GC-resident Tfh cells (GC-Tfh) express the G-protein coupled receptor S1PR2, which restrains their location to the GC ^36, 37^. Most GC-Tfh cells are located in the light zone – some forming an outer ring in the periphery, with a small proportion observed in the dark zone ^38, 39, 40, 41^. Germinal centre B cells present antigen to GC-Tfh cells which enables antigen-specific T and B cell to interact, and for T cells to deliver “help” in the form of CD40L and IL-21 ^32, 42, 43, 44, 45^. These signals “refuel” GC B cells by turning on expression of Myc, creating a metabolic reservoir in selected GC B cells that supports their re-entry into cell cycle and move again towards the dark zone, in what is known as “recycling”^46^. Such recycling is important to undergo further rounds of somatic hypermutation. Subsequent inertial cell cycling in the dark zone is supported by Foxo1, BATF, and Cyclin D3 ^46, 47, 48, 49^. The key role for GC-Tfh cells is thus to support the proliferation and recycling of GC B cells, with BCR signalling being crucial for GC B cell survival ^44, 45, 50, 51^ (Figure 3).

In addition to supporting the proliferation of germinal centre B cells, Tfh cells contribute to fine-tuning the specificity and affinity of the BCR repertoire of cells emerging from germinal centre - antibody secreting cells and memory B cells. In the current model of germinal centre selection, B cells with higher affinity BCRs can acquire more antigen from FDCs and outcompete lower affinity B cells for Tfh cell help by presenting more peptide:MHCII complexes ^52, 53^. This model is supported by experiments in which artificially enhancing peptide:MHCII presentation on lower-affinity B cells enables them to successfully engage Tfh cells and receive help ^54^. It is further supported by experiments in which acute depletion of over 90% of Tfh cells from established germinal centres results in the selective persistence of high affinity B cells^51^. In germinal centres under less stringent selection pressure – as seen in mice haploinsufficient for MHCII genes, or in which Tfh cell to GC B cell ratio is slightly reduced — alterations in affinity maturation are not observed ^51,55^.

Thus, the emerging view is that although Tfh cells can exert strong selective pressure on the germinal centre B cell receptor repertoire, the mechanisms may not follow a linear trend. Indeed, selection of GC B cells with a broad range of affinities has been widely reported, this affinity-permissive selection means that significant numbers of low-affinity B cells can exist alongside higher affinity cells, including members of the same clone that also contain high-affinity B cells ^50, 56, 57, 58, 59, 60, 61^. This may be important for preserving the diversity of response whilst simultaneously ensuring progressive affinity maturation ^57, 59, 62^. To reconcile this evidence of affinity-permissive selection in GC and the phenomenon of affinity maturation, a model has been proposed whereby plasma cell precursors expressing high-affinity antibodies receive higher levels of Tfh cell-derived help, especially secreted IL-21, resulting in division at higher rates compared with their lower-affinity counterparts ^63^. Consistent with this, IL-21 signaling is desensitized in GC B cells, and it regains sensitivity at the plasma cell precursor stage ^42, 45^. Another explanation for the described late emergence of low-affinity B cells during germinal center reactions involves a distortion in clonal phylogeny interpretation referred to as the “pull of the present”: low-affinity cells appear predominantly near the time of sampling because they have not yet been eliminated by selection, leading to an overrepresentation of these cells in late-stage phylogenies ^64^. Taken together, Tfh cells are part of an imperfect selection process that shapes the repertoire of B cells that emerge from the germinal centre.

Tfh cell responses are dynamic with a continual flux of T cells in and out of the germinal centre, enabling the responding T cells to evolve as the germinal centre reaction progresses. Changes in the composition of the Tfh cell population and the molecules they produce may contribute to germinal centre shutdown ^65, 66, 67^. In certain models, GC-Tfh cells shift from an IL-21 bias to predominantly IL-4 secretion which may contribute to germinal centre shut down by repressing Bcl6 expression ^68, 69, 70^. Suppression by Foxp3+ T cells also increases over time with T follicular regulatory (Tfr) cells accumulating in the GC and some GC-Tfh cells turning on Foxp3 thereby limiting helper cell function ^66, 71, 72, 73^. The resolution of GCs marks the end of Tfh cell activity in response to a particular antigenic encounter, while the generation of Tfh memory cells at any stage of differentiation creates a reservoir of both circulating and tissue resident cells that can rapidly support humoral immunity in response to rechallenge ^31, 51, 74, 75, 76^.

### Decoding Tfh cell differentiation in humans

In most human studies, it is not practical to sample Tfh cells directly from secondary lymphoid organs, with fine needle aspirates of lymph nodes and adenoid sampling reported in only a small number of studies ^76, 77, 78, 79, 80^. Studies directly assessing human lymph node Tfh cells after vaccination reveal a phenotypically diverse population, reflecting many of the Tfh cells subtypes reported in mice, as well as clonal expansion and metabolic shifts that evolve throughout the response ^13, 81, 82, 83, 84^. In the absence of direct sampling of human Tfh cells from lymphoid organs, circulating Tfh (cTfh) cells have been widely used as a proxy to infer aspects of Tfh cell biology in humans.

Circulating Tfh cells were first described as an expanded circulating T cell population in autoimmune disease in both mouse and humans ^85^, and were subsequently shown to expand after vaccination and have a superior B cell helper capacity when compared to other helper T cell subsets ^31, 86, 87, 88, 89^. cTfh cells are clonally, transcriptionally and epigenetically similar to those found in secondary lymphoid organs in humans ^81, 90, 91, 92, 93^. Analysis of paired blood and lymph node samples from human vaccination studies demonstrated that the circulating and tissue resident Tfh cells have distinct kinetics: cTfh frequencies peak in the first week after vaccination then return to baseline, while lymph node Tfh cells remain expanded for months ^31, 78, 81, 83^. Thus, while cTfh cells offer a valuable tool to understand Tfh cell biology in people, the timepoint post-challenge is a critical consideration when using them as biomarkers of responses in secondary lymphoid organs.

Much of our understanding of Tfh cell development in human comes from studies on individuals with monogenic mutations that cause inborn errors in immunity. These mutations primarily affect surface receptors, their ligands, and the downstream molecules involved in their signalling ^94^. People living with deficiencies in *ICOS/ICOSL* or *CD40L* have autosomal recessive combined immunodeficiency or X-linked hyper-IgM syndrome, respectively. Both are characterised by a marked reduction in cTfh cells and impaired humoral immunity ^94^, like mice deficient in the same genes. By contrast, CD28 deficiency in humans appears to have minimal impact on cTfh cells despite CD28 being essential for the formation and maintenance of Tfh cells in mice ^95^. Humoral immunity also remains largely unaffected in CD28-deficient humans, as evidenced by intact memory B cell generation, immunoglobulin class switching, and serum levels of antigen-specific IgG ^96^. This species-specific difference may be due to the ability of other co-stimulatory receptors to compensate for CD28 when their expression is sufficiently high, a mechanism that has been reported in mice ^97^.

Loss-of-function mutations in *SH2D1A*, which encodes SLAM-associated protein (SAP) that functions as an adaptor molecule for signalling lymphocytic activation molecule family receptors, cause X-linked lymphoproliferative disease (XLP1). Individuals with XLP1 have detectable cTfh cells, but fail to generate GC-Tfh cells and mount effective humoral immunity, indicating that cTfh cells predominantly arise at a precursor stage before the maturation of GC-Tfh cells ^31^. In contrast, patients with deficiency of the PD-1 immunosuppressive checkpoint receptor have an expanded cTfh cell population but impaired production of IL-21, which likely underlies their poor humoral responses upon vaccination. These phenotypes are largely recapitulated in mice deficient for PD-1, indicating a conserved role for PD-1 in regulating Tfh cells across species ^98^.

Several cytokines play essential roles in promoting human Tfh cell differentiation, evidenced by the reduction of cTfh cells in individuals carrying loss-of-function mutations in *IL21, IL21R, IL6ST*, and *IL6R*. These cytokine pathways activate the transcription factor STAT3, which is crucial for Tfh differentiation. Consequently, dominant-negative mutations in *STAT3* cause autosomal dominant hyper-IgE syndrome, leading to a lack of cTfh cells and compromised humoral immunity. A similar phenotype was observed in individuals with homozygous mutations in *ZNF341*, a transcription factor that regulates STAT3 expression and activation ^94^. Conversely, gain-of-function mutations in *STAT1* impairs Tfh cell function. These mutations in *STAT3* or *STAT1* specifically result in the loss of type 17 Tfh cells and the generation of a type 1-biased Tfh cell response ^94^.

Inborn errors of immunity can also cause excessive Tfh cell generation, as observed in hyperactivating mutations in PI3Kδ, leading to aberrant integration of signals downstream of ICOS and increased Tfh cell differentiation ^99, 100^. In one case study, a homozygous recessive mutation in ROQUIN-1 was associated with severe hyperinflammation and enhanced expression of Tfh markers ^101^. Together, inborn errors of immunity have identified both conserved and distinct mechanisms of Tfh cell formation in humans and mice that can directly inform relevant pathways that can be used to target Tfh cells in humans.

### Modulating Tfh cells in disease

Given their central role in regulating humoral immunity and secreting immunoregulatory cytokines such as IL-21 and CXCL13, Tfh cells are implicated in a wide range of pathological processes, including infectious diseases, autoimmunity, cancer and allergy. These roles have been extensively reviewed previously ^17, 95, 102, 103^. This section provides an updated synthesis of recent findings on the contributions of Tfh cells to these diseases and explores emerging strategies to target Tfh cells to limit disease symptoms.

### Boosting Tfh cells in infectious disease and vaccination

Tfh cells have a well described role in promoting immune heath. Work in genetically modified mice that either lack Tfh cells, or key Tfh cell molecules, has laid the foundation for understanding when Tfh cells are essential - and when they are dispensable - for the generation of protective humoral immunity. Following infection or immunisation, mice lacking Tfh cells or exhibiting impaired Tfh cell function, show reduced serum IgG, with the IgG1 class being more affected that other isotypes. Impaired Tfh cell function results in poor germinal centres, limited somatic hypermutation, affinity maturation and the longevity of the antibody response ^27, 104, 105, 106^.

**Figure 4:**
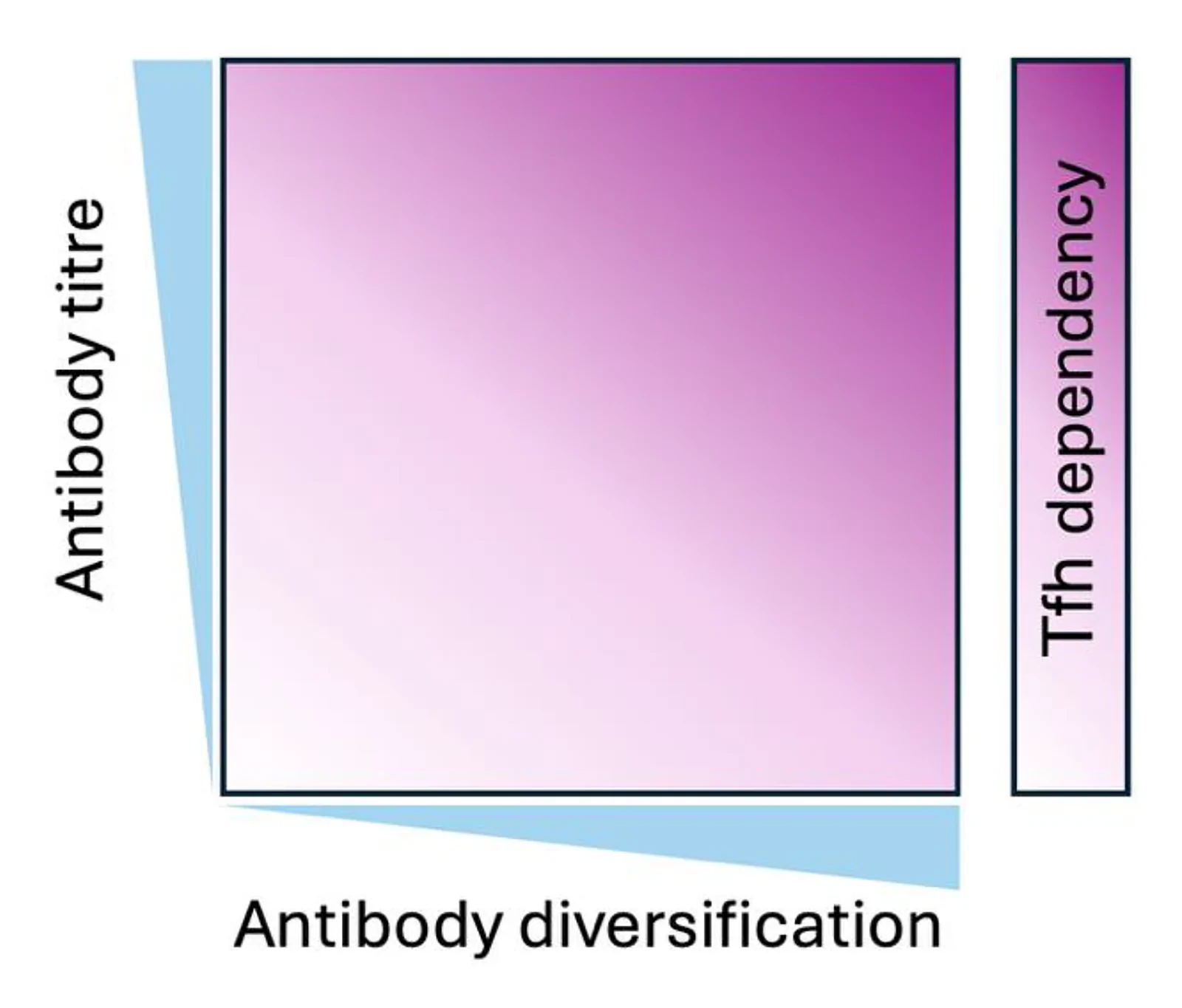
Tfh cells support humoral immunity to infections. Tfh cells become increasingly important for supporting anti-pathogen immunity when high titre and/or somatically mutated antibody responses are required.

How Tfh cells impact protective immunity depends on the pathogen and its dose. Mice that lack Tfh cells can generate protective immunity to low dose, but not high dose, influenza A virus infections ^104, 106^. Likewise, Tfh-deficient K18-hACE2 mice, that are permissive to SARS-CoV-2 infection, can generate protective immunity to subsequent SARS-CoV-2 infections, provided that antibodies reach a sufficient threshold ^107^. This is consistent with somatic hypermutation not being required to form SARS-CoV-2 neutralising antibodies in humans ^108^. By contrast, the formation of neutralising antibodies to vesicular stomatitis virus requires both germinal centres and Tfh cells ^109^. This is also the case in chronic lymphocytic choriomeningitis virus infection where Tfh cell dependent evolution of the antibody response is required to ultimately clear the virus ^105, 110, 111^. This is akin to the requirement for extensive somatic hypermutation to generate broadly neutralising antibodies to human immunodeficiency virus ^112^. Together these findings suggest that targeting Tfh cells may be a critical strategy to improve immunity to foreign pathogens when high-titre and/or somatically mutated antibody responses are required (Figure 4).

Vaccination remains one of the most cost-effective ways to promote human health. Most effective vaccines work by generating antibody responses that limit pathogen spread and reduce the severity of clinical disease. The wide array of vaccine platforms generated and tested during the COVID-19 pandemic highlighted that the requirement of Tfh cells for generating effective humoral immunity varies depending on vaccine format. In all vaccine formats Tfh-deficient mice had reduced spike-specific IgG titres, but adenoviral vector and protein/adjuvant vaccines had a greater dependence on Tfh cells for humoral immunity and memory B cell responses compared to mRNA vaccines ^107, 113, 114^. This occurs despite the lipid nanoparticles and their encapsulated mRNA stimulating potent Tfh cell responses and prolonged GCs ^115, 116^, suggesting that these vaccines stimulate multiple helper T cell subsets simultaneously to support antibody mediated immunity. Indeed, human vaccination studies show that different types of T cell responses positively correlate with humoral immunity in response to different vaccine formats; with IL-2 and TNF production linked with enhanced memory B cell numbers in protein/adjuvant vaccines, but not in mRNA vaccination ^117^. This highlights that the choice of vaccine format offers a tractable means of directing the T cell response towards Tfh-dependent humoral immunity.

The majority of vaccines that are currently given to adults elicit a recall response to previously encountered pathogens, such as influenza virus, SARS-CoV-2, or respiratory syncytial virus ^118, 119, 120^. While pre-existing immune memory enables a faster recall response, it can also create challenges for generating high titre antibody responses upon revaccination. These include vaccine antigen masking due to pre-existing antibodies, recall responses favouring short lived antibody responses, and the heterogeneity of the memory cell population influencing the quality of the boosted immune response ^121, 122, 123^. There is evidence that some of these challenges can be overcome by enhancing Tfh cell function during the recall response ^60^, highlighting Tfh cell targeting as a mechanism to enhance responses to revaccination. Tfh cell memory is generated during primary responses, therefore vaccine strategies that facilitate enhanced Tfh cell numbers may bolster recall responses to booster vaccines, including promoting Tfh17-skewed Tfh cell differentiation ^124^.

To date, modulating the adjuvant used in protein-subunit vaccines has been the most tractable way to enhance Tfh cell formation. TLR stimulating molecules are a prominent class of Tfh-promoting adjuvants ^90, 125, 126, 127, 128, 129^, as are adjuvants or vaccination strategies that enhance antigen access to the lymph node ^130, 131^. Adjuvants that stimulate mucosal-associated invariant T (MAIT) cells can indirectly boost Tfh cell numbers, highlighting the potential of mucosal vaccination strategies to support and amplify Tfh cell responses ^132, 133^. Likewise, promoting natural killer T cells can support Tfh generation during viral infection^134^. Inclusion of cytokine adjuvants formulated in lipid nanoparticle mRNA vaccines can also enhance humoral immunity, highlighting the tractability of modifying immunogenicity by targeting key pathways ^135^. Antigen design modifications aimed at enhancing T cell help have been shown to support enhanced humoral immunity in tonsil organoids and in mouse models ^136^. Notably, novel antigens designed to harness Tfh cell help are currently in clinical trials (NCT06810934).

### Limiting Tfh cell function in Autoimmune Diseases

Tfh cells have been implicated in autoimmunity when self-tolerance is broken via alterations in self-antigen presentation, or inappropriate co-simulation leading to activation of self-reactive T cells ^137, 138, 139, 140^. Historically, Tfh cells were thought to primarily promote affinity-matured autoantibody production via GCs in autoimmune diseases. However, this paradigm has evolved with the identification of Tph cells within tertiary lymphoid structures in inflamed non-lymphoid tissues, which facilitate local autoantibody production (reviewed in ^141^). Emerging evidence further highlights the role of splenic extrafollicular B helper T (eTh) cells - CD4^+^ PD-1^hi^ CXCR5^-^ CXCR3^+^ that are typically IL-10 producing - in supporting the development of pathogenic B cells outside of germinal centres. For example, preventing GC formation in mice carrying TLR7 gain-of-function mutations found in human lupus patients failed to reduce autoantibody production and halt disease progression. This implicated non-germinal centre pathways as critical drivers of B cell-driven autoimmunity ^138, 142^. Recently, IL-10-producing T cells – referred to as Th10 – have been shown to provide help to B cells in human lupus ^143^. A central question that emerges is whether Tph, eTh/Th10, and Tfh subsets in autoimmune diseases arise through distinct differentiation pathways, or whether they originate from a shared BCL6^+^ precursor population. Current evidence suggests they arise from overlapping differentiation pathways, rather than belonging to distinct lineages. Type I interferon – a cytokine central to SLE immunopathogenesis and a therapeutic target – enhances Tfh, eTh/Th10, and Tph differentiation, at least in part by inducing CXCL13 production ^144^. Depletion of PD-1 expressing cells, including Tfh and Tph, using PD-1-targetting chimeric antigen receptor T and NK cells resulted in improvements of disease in a mouse model of lupus, indicating the benefit of targeting these cells in autoimmune disease ^145, 146^.

Monoclonal antibodies that block key Tfh molecules including IL-21/IL-21R, ICOS/ICOSL and CD40 have been tested in clinical trials (Table 1) for various autoimmune diseases where the goal has been to limit Tfh cell abundance or function. ICOS is essential for Tfh cell formation and motility^147^, and blocking this co-stimulatory receptor or its ligand in people is associated with diminished numbers of Tfh cells. Early phase clinical trials targeting this costimulatory receptor showed improvement of disease symptoms ^148^. The canonical Tfh cell cytokine, IL-21, has been directly targeted in multiple autoimmune diseases with blocking antibodies, either alone or in combination with other treatments (Table 1). In type 1 diabetes, combined treatment with anti-IL-21 and a glucagon-like peptide-1 (GLP-1) receptor agonist, preserved β-cell function to a similar extent as other treatments, but with a superior safety profile ^149^. By contrast, an anti-CD40 monoclonal antibody did not have a significant impact on kidney function in lupus nephritis patients ^150, 151^. Low dose IL-2 treatment has been successful in reducing Tfh cells in systemic lupus erythematosus patients by, which is linked with reduced disease activity ^152^. These trials highlight the therapeutic potential of targeting Tfh cells in the many human autoimmune diseases in which they are implicated.

### Targeting Tfh cells in cancer

The critical role of CD4^+^ T cells in bolstering CD8^+^ T cell effector function was first described in the 1980s ^153^. Because of the persistent nature of cancer, CD8 T cells need to maintain functionality while avoiding exhaustion. This is facilitated by a subset of TCF1^+^ stem-like precursor CD8^+^ T cells that, despite expressing markers of exhaustion, retains the capacity for self-renewal and differentiation into cytotoxic effector cells ^154^. The maintenance and differentiation of these CD8^+^ T cells into effector cells depend on IL-21 secreted by CD4^+^ T cells, with Tfh cells identified as a key source of this cytokine ^155, 156^. These findings in mouse models align with earlier immunophenotyping studies in human tumours, where Tfh cells signatures and their effector molecules, including IL-21 and CXCL13, correlated with favourable prognosis ^157, 158^. The PD-1 axis is routinely targeted in cancer immunotherapy to enhance CD8 T cell responses and promote anti-tumour immunity. Mechanistically, anti-PD-1 treatment was found to enhance IL-4 secretion from Tfh cells, which in turn supported CD8 responses against tumours, highlighting a key role for Tfh cells in promoting antitumor cellular immunity ^159^.

Beyond supporting CD8 T cells, CXCL13 production from Tfh cells can also contribute to the formation of tumour-associated tertiary lymphoid structures, a hallmark of favourable clinical outcomes, including enhanced responses to immunotherapy (reviewed in ^160, 161^). A critical gap remains in understanding the interplay between Tfh cells and tumour-reactive antibodies. Emerging evidence suggests tumour-reactive antibodies improve prognosis ^162, 163^, yet the mechanisms linking Tfh cells to antibody-mediated anti-tumour immunity are poorly understood and may represent a new avenue for novel therapeutic strategies.

Tfh cells can also be the source of tumor cells in a group of cancers described as nodal T follicular helper cell lymphomas ^160^. An ICOS targeting monoclonal antibody has shown cTfh cell depleting capability in a phase 1 clinical trial ^164^ (Table 1), with larger trials required to determine clinical impact on disease.

### Limiting Tfh cell function in Allergic & Atopic Disease

Tfh cells, particularly the IL-4-producing Tfh2 subset whose differentiation is initiated by type I IFN-activated dendritic cells, are pivotal in promoting IgE production in allergic diseases ^168, 169, 170^. Another distinct Tfh subset, Tfh13, co-expresses and supports high-affinity, anaphylactic IgE production as well as allergen reactive GCs in an IL-13 and IL-21 dependent manner ^171, 172^. Consistent with a similar role in humans, Tfh13 cells are enriched in patients with food or aeroallergen sensitivities ^172^, and the abundance of allergen-specific cTfh cells can be reduced by allergen immunotherapy ^173, 174^. In mice, the frequency of Tfh13 cells and symptoms of asthma can be modified by dietary supplementation that supports microbial production of butyrate ^175^, highlighting dietary interventions as a potential avenue for treatment. Treatment with Dupilumab – a human monoclonal antibody targeting IL-4 and IL-13 signalling – has revolutionised allergic and atopic disease management. It is associated with an alteration in cTfh phenotypes skewing towards cTfh17 cells and IL-2 production, alongside reduction in conventional Th2 cells ^176, 177^. This highlights the plasticity of T cells and shows that reducing the quantity and modifying the phenotype of Tfh cells in allergic and atopic disease correlates with improved health outcomes.

### Ageing and Tfh cells

Ageing is the biggest risk factor for disease, infectious or otherwise. Any attempt to therapeutically modulate Tfh driven disease processes or support robust vaccine responses must take the age-dependent changes of these cells into account. There is evidence that it is possible to modulate Tfh cells in ageing to compensate for changes that are linked with poor function ^178^. Much of the insight into how human Tfh cells change with age comes from cTfh cells. The abundance of cTfh cells reduced in adults over 65 years of age, compared with adults under 40 years, and is associated with lower titre antibody responses in older vaccinees ^89, 118, 125, 179^.

Gene expression profiling showed that cTfh cells from older donors sense increased inflammation, including the TNF-NFκB and IL-2-STAT5 pathways, both of which are known to suppress Tfh cell formation ^118, 179, 180, 181^. Mouse studies support a functional role for increased inflammation in limiting Tfh cell responses, with T cells from younger donors having reduced Bcl6 expression and failing to undergo full differentiation into GC-Tfh cells in an aged microenvironment ^182, 183^. Indeed, suppressing this inflammation prior to vaccination can improve vaccination responses in older adults ^184^.

Murine adoptive transfer experiments provided a causal link between ageing-related changes in Tfh cells and poor humoral immunity. T cells from aged (>20month old) donors were less able to support high quality humoral immune responses ^185, 186, 187, 188^. Notably, this impairment occurs despite comparable densities of Tfh cells within GCs between young and aged mice ^189, 190^ and in younger and aged macaques ^191^. This finding aligns with data from human adenoid samples, where a positive correlation of Tfh cell with GC B cell numbers persists, despite an age-dependent decline in the GC response ^79^. CD40L and IL-4 expression has been reported to be reduced on T cells from older mice and humans, while IL-10 is increased ^182, 186, 188, 192, 193^, possibly limiting their helper function on a per cell basis. Blocking IL-10R signalling in ageing can increase antibody production^193^, suggesting this cytokine limits humoral immunity in ageing^193^, although IL-10 can support antibody production in other contexts^194^. *Ex vivo* Tfh:B cell co-culture assays have reported both intact and diminished helper cell function with age ^89, 187, 195^, indicating that other aspects of Tfh biology, such as enrichment in the dark zone of old mice ^189^ contribute to impaired T cell help in ageing. The use of adjuvants, cytokines and checkpoint blockade has enhanced Tfh cell responses in aged mice and macaques, demonstrating that boosting Tfh cells in ageing is a feasible and effective strategy to support vaccine responses ^187, 191, 196^.

Targeting Tfh cells specifically in older people will require the combined insights from both immunology and geroscience. Whilst current approaches to boost Tfh cell responses in vaccines focus on pushing the immune system harder with adjuvants or high antigen dosing ^178^, there is evidence that limiting the higher levels of inflammation in older adults prior to vaccination may be beneficial ^197^. This might be achieved by straightforward interventions such as oral Vitamin D3 supplementation prior to vaccination ^198, 199^. Studies in older people have linked high expression of CD39 with poor Tfh cell formation during ageing. Inhibition of this ecto-NTPDase receptor or its downstream purinergic signalling enhanced Tfh cell numbers in mice, with genetic variants in humans demonstrating a similar mechanism ^200^. This underscores that determining how Tfh cell responses evolve across the lifespan can reveal novel targets for therapeutic intervention.

Targeting age-dependent changes in metabolism offers a promising avenue for modulating Tfh cells in ageing, and will be discussed in depth in the next section. In the context of ageing, treatment with metformin, a blood glucose regulator and candidate anti-ageing drug ^201^, enhanced cTfh cell responses in older adults after seasonal influenza vaccination although this was not accompanied by an increase in serum antibodies ^202^. The metabolic and immunologic changes associated with GLP-1 receptor agonists indicate that this axis may also represent a potential modulator of vaccine responses in ageing ^203, 204^, and support investigation of metabolic interventions to promote Tfh cells in the context of healthy ageing.

### Systemic metabolism and Tfh cells

In addition to older people, other sectors of our society, including malnourished individuals and people with high body mass index, exhibit diminished vaccine responses and efficacy ^178, 205^. Recent studies have revealed various metabolic pathway that regulate Tfh function and vaccine responses. Major metabolic pathways, including glycolysis, mitochondrial oxidative phosphorylation (OXPHOS), and fatty acid oxidation, are fundamental to meeting the energy and substrate demands of T cell homeostasis. They also direct the programming required during T cell activation, proliferation, and the transition from effector to memory states. Essential metabolites such as glucose, amino acids, and lipids not only serve as building blocks in these pathways but they also act as signalling molecules, initiating specific gene regulation in T cells ^206^. Although these metabolic processes are common across most CD4^+^ T cell subsets, including Tfh cells, distinct metabolic preferences by different T cell subsets create opportunities for targeted modulation of Tfh cell function. Tfh-specific metabolic targets have been identified using CRISPR screening approaches, with the potential to support target identification in further interventional studies^207, 208^.

The mammalian target of rapamycin (mTOR) is a well-established signalling hub, integrating cues from many external stimuli leading to changes in metabolic function ^209^. mTOR interacts with different adaptor proteins to form two complexes: mTOR complex 1 (mTORC1) and mTOR complex 2 (mTORC2). mTORC1 plays a pivotal role in promoting cell cycle entry and metabolic reprogramming to support early T cell activation, while mTORC2 is crucial for memory T cell maintenance and function ^210^. Both mTORC1 and mTORC2 signaling pathways are essential for optimal Tfh cell number and function, as demonstrated by the reduction in Tfh cells and diminished humoral immunity observed in mice with T cell-specific deletions of Raptor or Rictor - defining components of mTORC1 and mTORC2 respectively ^211^. mTORC1 also promotes the generation and suppressive function of Tfr cells ^212^. Despite this key role for mTOR signaling in Tfh cell generation, low dose mTORC1 inhibition improves antibody responses to influenza vaccination in older adults ^213^, possibly by limiting the increased inflammation associated with age. Together, this highlights the complexity of targeting highly conserved pathways that have extensive roles across cell types and systems.

mTORC1 plays a central role in promoting glycolysis in T cells by upregulating the expression of glucose transporter 1 and other glycolytic enzymes. The balance between glycolysis and OXPHOS in the generation of Tfh cells varies depending on experimental models, as summarized elsewhere ^214^. In brief, although glycolysis is required for Tfh cell generation, excessive glycolysis may favour Th1 differentiation over Tfh differentiation, partly through the production of IL-2 or enhanced IL-2 signaling ^215^. In contrast, autoreactive Tfh cells in several mouse models of lupus exhibit a preference for glycolysis. Treatment with 2-deoxy-D-glucose (2DG), a non-metabolizable glucose analogue, reduced both autoreactive Tfh cells and autoantibodies in lupus-prone mice ^216^, demonstrating that targeting glycolysis is a tractable way to target Tfh cells.

The generation and survival of Tfh cells is regulated by two distinct cell death pathways, ferroptosis and pyroptosis, which both involve metabolic regulation in a context-dependent manner ^217^. Lymph node Tfh cells are susceptible to ferroptosis and their survival depends heavily on glutathione peroxidase 4 (GPX4), the primary enzyme responsible for scavenging cellular lipid ROS ^218^. This dependency for Tfh cell survival on GPX4 can be rescued in mice by modifying dietary lipid composition to reduce lipid ROS accumulation within Tfh cells^219^. In contrast, Tfh cells located in the gut and non-lymphoid tissues are more vulnerable to pyroptosis, a process mediated by the activation of purinergic receptor P2X7R in response to excessive extracellular ATP ^220, 221^. This knowledge enables strategies to enhance Tfh cell function following vaccination. For example, dietary selenium supplementation has been shown to boost the expression of the selenoenzyme glutathione peroxidase 4 ^218^, while altering dietary fatty acid contents may limit ferroptosis, thereby promoting Tfh cell function and improving antibody responses after vaccination^219^.

Another metabolic hormone, leptin, promotes Tfh cell differentiation and enhances vaccine responses ^222^. Furthermore, phosphatidylethanolamine synthesis plays a key role in regulating cell surface CXCR5 expression, thereby further influencing Tfh cell function ^208^. Moving forward, an integrated, systematic approach is required to study how the interplay between transcriptomes, proteomes, and metabolomes contributes to the overall fitness of Tfh cells, particularly in diverse human populations. Understanding these interactions will be critical to developing strategies that improve vaccine responses and addressing the challenges posed by metabolic dysregulation.

### Future prospects for targeting Tfh cells

The biology of Tfh cells can be influenced by intrinsic and extrinsic cues including genetic, transcriptional, translational and metabolic factors. Monoclonal antibodies and vaccines that specifically aim to modulate Tfh cells are already being tested in clinical trials, with some promise. Identification of new targets to modulate Tfh cell biology will require new innovations in fundamental bioscience. One of the key bottlenecks in identifying new targets to modulate Tfh cell biology has been the low abundance of these cells relative to the sensitivity of laboratory techniques to perform unbiased analyses on Tfh cells in various disease states. Single cell nucleic acid analysis methods have advanced our understanding of Tfh cell biology and identified mechanisms through which Tfh cell biology could be modulated to promote health ^82, 223, 224, 225, 226, 227^. However, unbiased assessment of proteomic and metabolic factors have, to date, relied on analysis of larger pools of Tfh cells which required expansion *ex vivo* prior to analysis ^211, 228^. Recent advances in spatial proteomics and metabolomics have enabled high-resolution analyses of Tfh cells *in situ* in tissues. However, the densely packed structure of secondary lymphoid organs means that disentangling the signal coming from Tfh cells from that of the surrounding B cells and follicular dendritic cells is a challenge ^229, 230^. New single cell analysis methods create opportunities to link changes in proteostasis and metabolism to Tfh cell number or function in human disease, particularly when the number of cells available for analysis are limited ^231, 232, 233^. Once key Tfh cell modulators are identified, it will be essential to validate their potential as therapeutic targets using human-relevant systems.

The anatomical location of Tfh cells within secondary lymphoid organs poses a significant challenge for functionally screening interventions aimed at targeting human Tfh cells. The discovery that human Tfh-like cells with B cell helper function can be generated *in vitro* via IL-12 - mirroring the cytokine’s role *in vivo* ^234, 235^, demonstrated the feasibility of using *in vitro* human cell approaches to study Tfh cells development, and to evaluate novel therapeutic interventions. High-content screening approaches have demonstrated that several cytokines, and combinations thereof, can support human Tfh cell differentiation *in vitro* ^236, 237^, with studies of Tfh cells in patients with inborn errors of immunity confirming the importance of these cytokines *in vivo* ^94^. To investigate human Tfh cell function in vitro, new organoid systems have been developed to model their function in response to vaccines and to also model Tfh cell derived follicular lymphoma ^238, 239, 240^. Indeed, *in vitro* testing has shown some promise as a method of screening potential approaches to boost human Tfh cell number and function ^241^. Tonsil organoids have been used to test multi-valent vaccine antigen approaches that recruit a more diverse range of helper T cells to support humoral immunity. These findings were confirmed in mice, highlighting the benefit of combining in vitro reductionist human systems with in vivo testing ^136^. The ability to modify T cells from human secondary lymphoid tissues with CRISPR-gene editing and assess their function in organoid systems ^242^ provides an opportunity to identify novel pathways and molecules that can target Tfh cell formation and function ^243^. Together, innovations in technology and in vitro biology combined with in vivo models is expected to fuel the next cycle of innovation in finding drugs that target Tfh cells.

## Figures and Tables

**Table 1:** Tfh cell targeting molecules in clinical trials in autoimmunity and cancer.

Treatment	Target	Disease	NCT number	Publication
AMG 557 (Prezalumab; anti-ICOSL)	ICOSL	Systemic lupus erythematosus with arthritis (Phase 1b) Primary Sjogren’s Syndrome (Phase 2a)	NCT01683695 NCT02334306	148
NNC0114-0005 (anti-IL-21)	IL-21	Rheumatoid arthritis (Phase 1) Type 1 Diabetes (Phase 2, combined with liraglutide)	NCT01208506 NCT02443155	165 149
BI 655064 (anti-CD40)	CD40	Healthy people (Phase 1) Lupus Nephritis (Phase 2)	NCT01510782 NCT02770170	166 150 151
BOS161721 (anti-IL-21)	IL-21	Healthy controls (Phase 1) Systemic Lupus Erythematosus (Phase 1b/2)	NCT03036865 NCT03371251	167
MEDI-570 (anti- ICOS)	ICOS	Nodal T follicular helper cell lymphomas (Phase 1)	NCT02520791	164
Low dose IL-2	IL-2R	Systemic lupus erythematosus (interventional)	NCT02084238	152
